# Nuclear Epidermal Growth Factor Receptor Overexpression as a Survival Predictor in Oral Squamous Cell Carcinoma

**DOI:** 10.3390/ijms24065816

**Published:** 2023-03-18

**Authors:** Marko Tarle, Marina Raguž, Danko Muller, Ivica Lukšić

**Affiliations:** 1 Department of Maxillofacial Surgery, Dubrava University Hospital, 10000 Zagreb, Croatia; tarlemarko1@gmail.com; 2 School of Dental Medicine, University of Zagreb, Gundulićeva 5, 10000 Zagreb, Croatia; 3 Department of Neurosurgery, Dubrava University Hospital, 10000 Zagreb, Croatia; 4 School of Medicine, Catholic University of Croatia, 10000 Zagreb, Croatia; 5 Department of Pathology and Cytology, Dubrava University Hospital, 10000 Zagreb, Croatia; 6 School of Medicine, University of Zagreb, 10000 Zagreb, Croatia

**Keywords:** squamous cell carcinoma, oral cavity, biomarkers, nEGFR, immunocytochemistry

## Abstract

The aim of this study was to determine, by immunohistochemical methods, the expression of nEGFR and markers of cell proliferation (Ki-67), cell cycle (mEGFR, p53, cyclin D1), and tumor stem cells (ABCG2) in 59 pathohistological samples of healthy oral mucosa, 50 oral premalignant changes (leukoplakia and erythroplakia), and 52 oral squamous cell carcinomas (OSCC). An increase in the expression of mEGFR and nEGFR was found with the development of the disease (*p* < 0.0001). In the group of patients with leukoplakia and erythroplakia, we found a positive correlation between nEGFR and Ki67, p53, cyclin D1, and mEGFR, whereas in the group of patients with OSCC, we found a positive correlation between nEGFR and Ki67, mEGFR (*p* < 0.05). Tumors without perineural (PNI) invasion had a higher expression of p53 protein than tumors with PNI (*p* = 0.02). Patients with OSCC and overexpression of nEGFR had shorter overall survival (*p* = 0.004). The results of this study suggest a potentially important independent role of nEGFR in oral carcinogenesis.

## 1. Introduction

Oral Squamous Cell Carcinoma (OSCC) is the most common malignant tumor of the head and neck. In Europe and the United States, it accounts for 2–3% of all malignancies [1]. In 2020, 377,713 people were diagnosed with lip cancer and OSCC worldwide, while 177,757 patients died, with a trend toward increasing numbers of patients under 50 years of age [2,3]. The large expansion of OSCC research and advances in diagnostic and therapeutic methods over the past 30 years have not resulted in a significant increase in the 5-year survival rate of patients, which is still about 55% [4]. Moreover, more than 40% of patients already have regional metastases at the time of disease diagnosis, and more than 60% of patients have tumors larger than 4 cm, indicating ineffective prevention of the disease. New strategies are needed to change the current uniform approach in treating all patients with the same clinical and pathohistologic features [5]. Treatment of patients should be based on proven biomarkers that provide the basis for individual differences in the genetic and biological behavior of tumors. Accumulation of mutations, chromosomal damage, and loss of cell control function result in histologic changes of normal oral epithelium into dysplasia, carcinoma in situ, and invasive OSCC [6]. Although the role of mEGFR in HNSCC is well established and numerous anti-EGFR drugs have been developed and are routinely used, poor response to therapy and resistance to therapy are frequently recorded, possibly due to the existence of nonclassical subcellular signaling of the Epidermal growth factor receptor (EGFR) pathway. Recent studies suggest that EGF, H2O2, UV radiation, therapeutic agents, and ionizing radiation may cause translocation of EGFR to the nucleus, where nuclear EGFR (nEGFR) interacts with various transcription factors (cyclin D1, ABCG2/BCRP, Aurora kinase A, COX-2, gene regulator c-Myc, iNOS) and acts on the activation of numerous genes involved in cell proliferation, tumor progression, and DNA repair [7,8,9,10]. Available literature indicates that overexpression of nEGFR in ovarian, breast, oropharyngeal, laryngeal, and esophageal cancers negatively affects disease prognosis and resistance to radiotherapy and chemotherapy, whereas its role in oral malignancies has not yet been investigated [8,11,12,13,14,15,16]. The above only confirms the complexity and scope of the network of signaling pathways mediated by EGFR activation that play an important role in cancer progression. The aim of this study is to use immunohistochemical methods to determine the expression of nEGFR in healthy oral mucosa, premalignant changes of the oral cavity (leukoplakia and erythroplakia), and OSCC, and to determine its influence on disease progression and clinical outcome in patients with OSCC. In addition, we analyzed the expression of markers of cell proliferation (Ki-67), cell cycle (mEGFR, p53, cyclin D1), and tumor stem cells (ABCG2) in the subjects’ samples, plus their correlation with nEGFR expression.

## 2. Results

We analyzed the expression of nEGFR and other observed biomarkers (Ki-67, p53, cyclin D1, mEGFR, ABCG2) by immunohistochemical methods in 161 subjects divided into three groups: 59 subjects with healthy oral mucosa, 50 patients with premalignant changes (31 leukoplakias and 19 erythroplakias), and 52 patients with OSCC in all TNM stages. The demographic data of the groups of subjects are shown in Table 1.

### 2.1. Results of Immunohistochemical Staining

#### 2.1.1. Expression of Ki-67 in Healthy Oral Mucosa, Premalignant Changes, and Invasive Oral Squamous Cell Carcinoma

**Figure 1:**
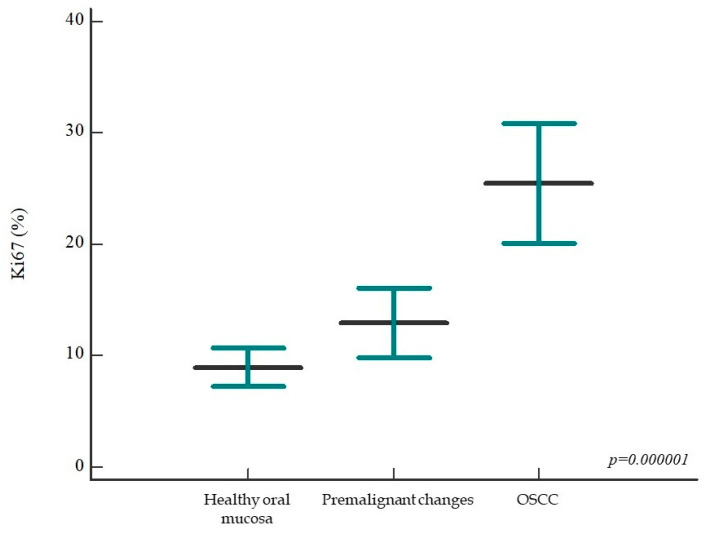
Percentage of Ki-67 proliferation index between analyzed patient groups. The percentage of Ki-67 proliferation index is significantly higher in the group of patients with OSCC than in individuals with healthy oral mucosa and premalignant changes (*p* = 0.000001); no statistically significant difference was found between the latter two groups. Horizontal lines indicate mean ± standard deviation; *p*, significance level in graph ANOVA.

**Figure 2:**
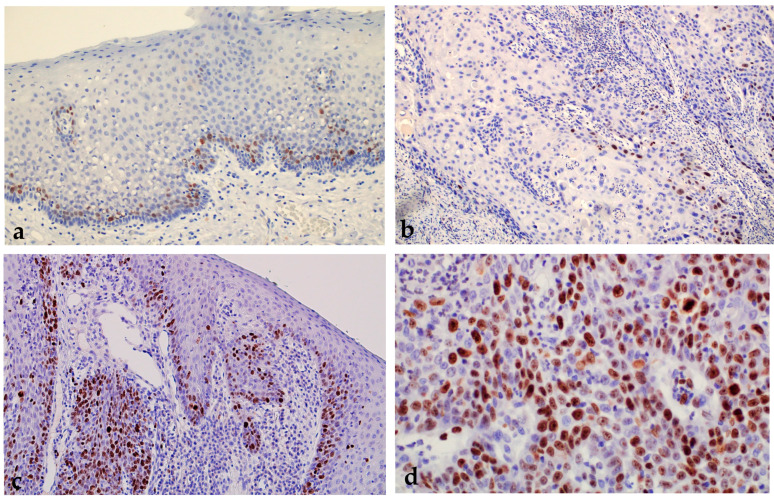
Oral erythroplakia with weak expression of Ki-67. Magnification 200× (**a**). OSCC showing a very low proliferation index. Magnification 100× (**b**). Immunohistochemical expression of Ki-67 in oral leukoplakia with moderate proliferation activity. Magnification 100× (**c**). OSCC with high proliferation activity with expression of Ki-67 in more than 30% of nuclei. Magnification 400× (**d**).

The percentage of the Ki-67 proliferation index in the studied groups ranged from 0% to 81% with a mean expression value of 15.51 ± 14.87%. As expected, the percentage of Ki-67 proliferation index expression in the group of patients with OSCC is significantly higher (mean value 25.46 ± 19.22%) than in the group with healthy oral mucosa (mean value 8.93 ± 6.68%) and in the group of patients with premalignant changes (13 ± 10.99%). A statistically significant difference in the expression of the Ki-67 proliferation index was found between subjects with healthy oral mucosa and premalignant changes on the one hand and patients with OSCC on the other (*p* = 0.000001) (Figure 1 and Figure 2).

In the group of patients with premalignant changes, when comparing the percentage of Ki-67 proliferation index in relation to the presence of oral epithelial dysplasias, a statistically significant higher percentage of Ki-67 proliferation index was observed in the subgroup of high-grade dysplasias (median 18.87% with a range of 6% to 30.5%) compared with low-grade dysplasias (median 10.34% with a range of 1 to 20.6%) (*p* = 0.005). 

#### 2.1.2. Expression of p53 in Healthy Oral Mucosa, Premalignant Changes, and Invasive Oral Squamous Cell Carcinoma

**Figure 3:**
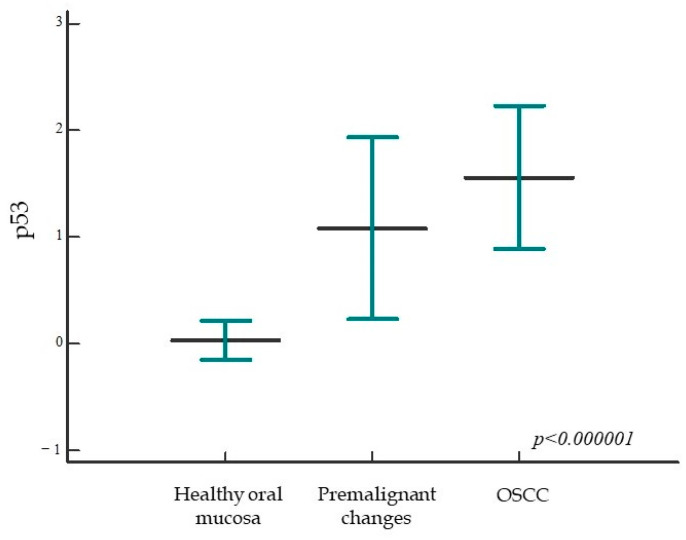
Expression of p53 protein in the studied groups. The percentage of p53 protein expression is significantly higher in the group of patients with premalignant changes and OSCC compared with subjects with healthy oral mucosa; moreover, a statistically significant difference was found between all studied groups (*p* < 0.000001). Horizontal lines indicate mean ± standard deviation; *p*, significance level is marked in Kruskal–Wallis graph.

**Figure 4:**
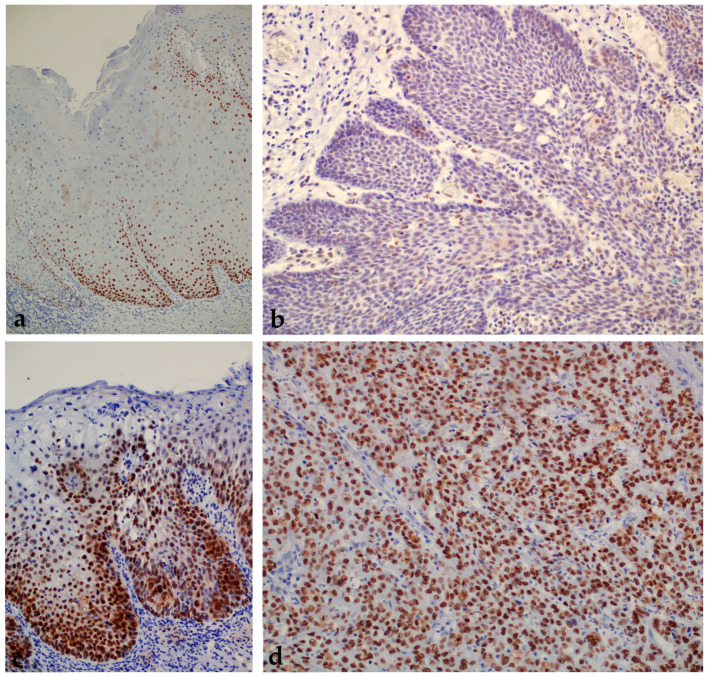
Moderate expression of p53 protein in oral leukoplakia according to the Allred scoring system (+). Magnification 100× (**a**). OSCC showing moderate p53 protein immunoreactivity according to the Allred scoring system (+). Magnification 200× (**b**). Oral erythroplakia with severe dysplasia and strong p53 protein expression in the dysplastic part of the affected epithelium according to the Allred scoring system (++). Magnification 200× (**c**). Strong immunohistochemical expression of p53 in OSCC according to the Allred scoring system (++). Magnification 200× (**d**).

When analyzing the expression of p53 protein, we found a statistically significant higher expression of the tested protein in the group of patients with premalignant changes and OSCC compared with the control group; also, a statistically significant difference was found between all analyzed groups (*p* < 0.000001) (Figure 3 and Figure 4). When comparing the percentage of p53 protein expression in the dysplasia group by subgroup, no statistically significant difference was found (*p* = 0.11).

#### 2.1.3. Expression of Cyclin D1 in Healthy Oral Mucosa, Premalignant Changes, and Invasive Oral Squamous Cell Carcinoma

**Figure 5:**
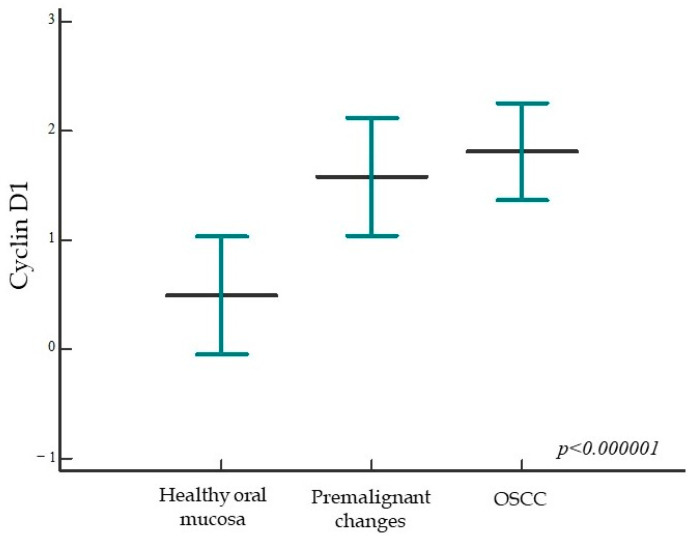
Cyclin D1 protein expression in the studied groups. The percentage of cyclin D1 protein expression is significantly higher in the group of patients with premalignant changes and OSCC compared with subjects with healthy oral mucosa; moreover, a statistically significant difference was found between all studied groups (*p* < 0.000001). Horizontal lines show mean ± standard deviation; *p*, significance level is marked on Kruskal–Wallis graph.

**Figure 6:**
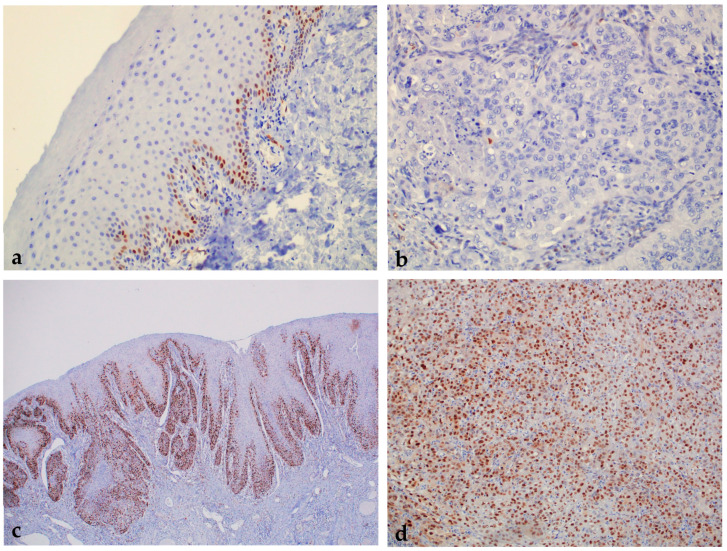
Oral leukoplakia with weak cyclin D1 protein expression according to the Allred scoring system (0). Magnification (**a**). OSCC without immunohistochemical expression of cyclin D1 according to the Allred scoring system (0). Magnification 200× (**b**). Strong immunohistochemical expression of cyclin D1 in oral leukoplakia with moderate dysplasia according to the Allred scoring system (++). Magnification 100× (**c**). OSCC with strong expression of cyclin D1 according to the Allred scoring system (++). Magnification 100× (**d**).

Analyzing the expression of cyclin D1, we found a statistically significant higher expression of the tested protein in the group of patients with premalignant changes and invasive OSCC compared to subjects with healthy oral mucosa; moreover, a statistically significant difference was found between all analyzed groups (*p* < 0.000001) (Figure 5 and Figure 6). When comparing the percentage of cyclin D1 expression in the dysplasia group by subgroups, no statistically significant difference was found (*p* = 0.18).

#### 2.1.4. ABCG2 Expression in Healthy Oral Mucosa, Premalignant Changes, and Invasive Oral Squamous Cell Carcinoma

**Figure 7:**
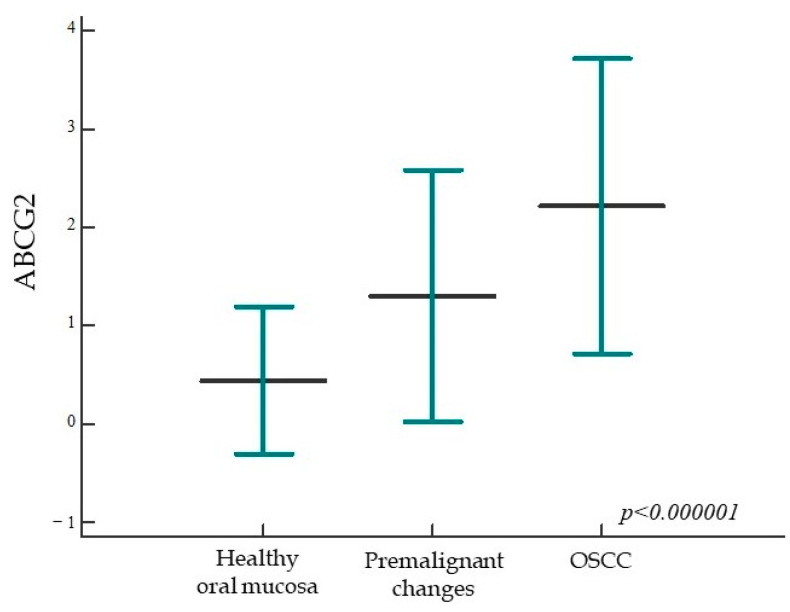
ABCG2 protein expression in the studied groups. The percentage of ABCG2 expression is significantly higher in the group of patients with premalignant changes and OSCC compared to subjects with healthy oral mucosa; moreover, a statistically significant difference was found between all studied groups (*p* < 0.000001). Horizontal lines indicate mean ± standard deviation; *p*, significance level is marked on Kruskal-Wallis graph.

**Figure 8:**
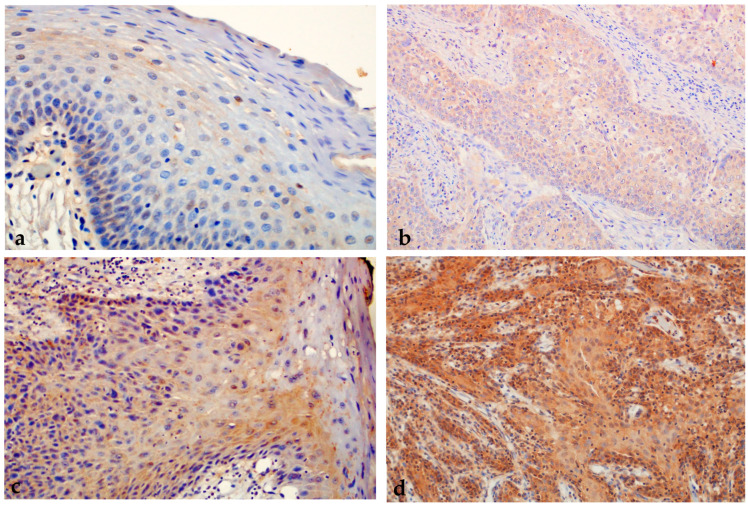
Moderate immunohistochemical expression of ABCG2 (++) in untransformed oral leukoplakia. Magnification 400× (**a**). Moderate immunohistochemical expression of ABCG2 (++) in OSCC. Magnification 200× (**b**). Strong immunohistochemical expression of ABCG2 (+++) in malignant transformed oral erythroplakia with severe epithelial dysplasia. Magnification 200× (**c**). Strong immunohistochemical expression of ABCG2 (+++) in OSCC. Magnification 200× (**d**).

Analyzing the expression of ABCG2, we found a statistically significant higher expression of the tested protein in the group of patients with premalignant changes and OSCC compared to subjects with healthy oral mucosa; moreover, a statistically significant difference was found between all analyzed groups (*p* < 0.000001) (Figure 7 and Figure 8). When comparing the percentage of ABCG2 expression in the group of dysplasias by subgroups, a statistically significant difference was found, i.e., ABCG2 expression is stronger in higher grade dysplasias (*p* = 0.02).

#### 2.1.5. Expression of nEGFR and mEGFR in Healthy Oral Mucosa, Premalignant Changes, and Invasive Oral Squamous Cell Carcinoma

**Figure 9:**
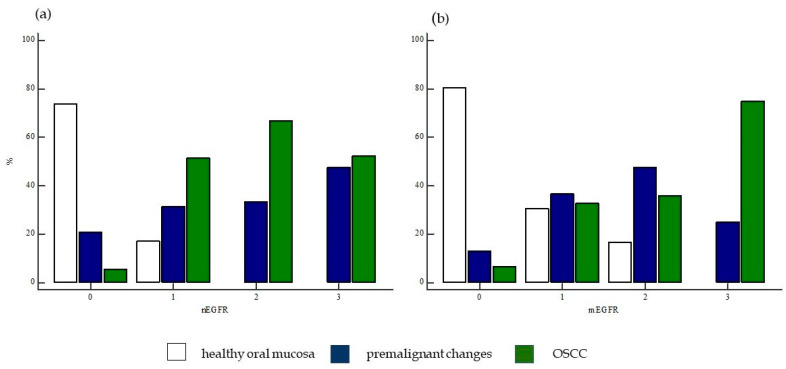
Expression of nEGFR and mEGFR between the studied groups of subjects. Comparisons of the expression of (**a**) nEGFR and (**b**) mEGFR between the groups of subjects analyzed showed statistical significance (χ^2^ = 85.96, *p* < 0.0001; χ^2^ = 70.40, *p* < 0.0001). Legend: nEGFR—nuclear EGFR; mEGFR—membrane EGFR; 0—negative, 1—weak expression, 2—moderate expression, 3—strong expression.

**Figure 10:**
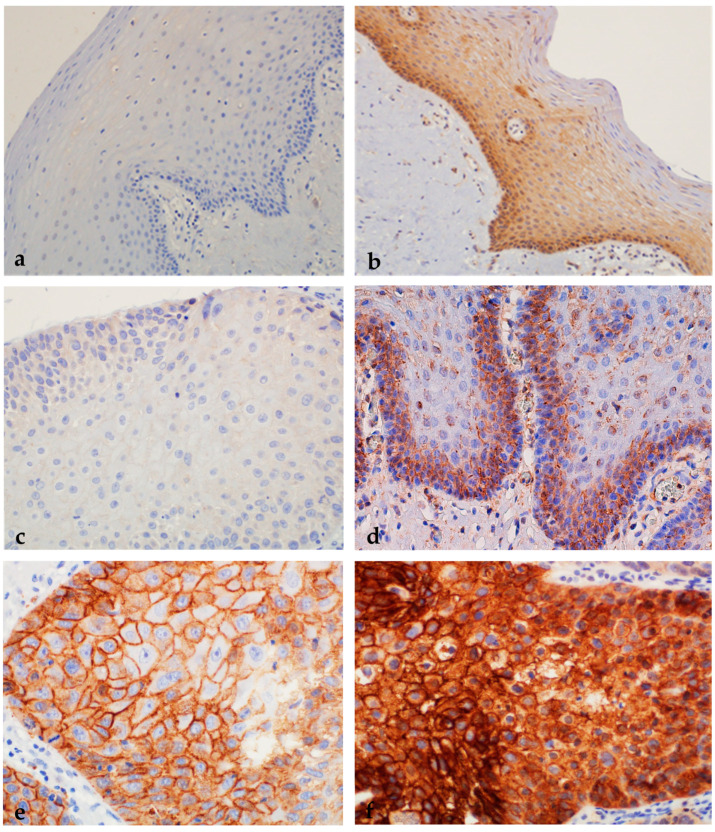
Immunohistochemical expression of mEGFR in healthy oral mucosa, oral premalignant changes, and OSCCs. (**a**) Negative expression of EGFR on the membrane of oral epithelium. (**b**) Moderate complete membrane staining of EGFR in oral mucosa (++). Magnification 200×. (**c**) Incomplete membrane staining of EGFR in more than 10% of cells in oral leukoplakia (+). (**d**) Moderate complete membrane staining of EGFR in oral leukoplakia (++). Magnification 400×. (**e**) Moderate complete membrane staining of EGFR in more than 10% of cells in OSCC (++). (**f**) Strong complete membrane staining in more than 10% of cells in OSCC (+++). Magnification 400×.

**Figure 11:**
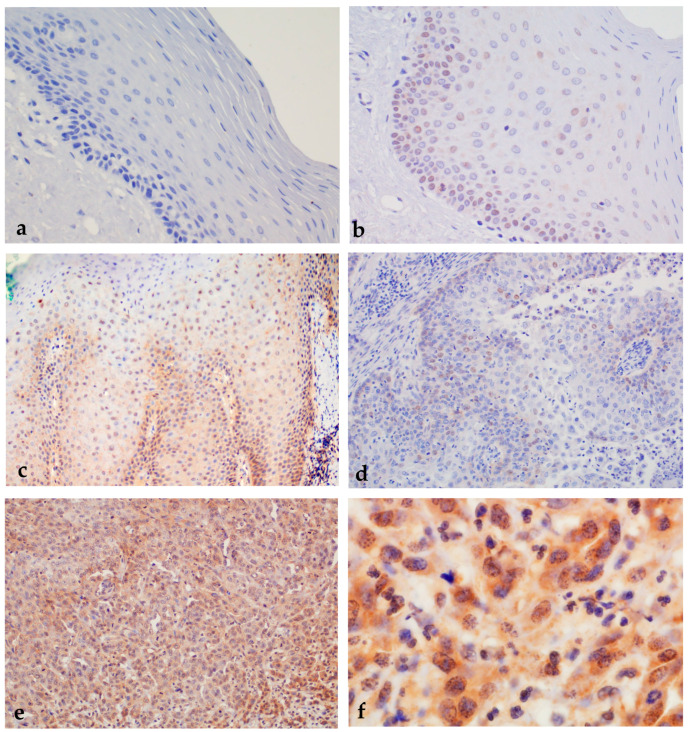
Immunohistochemical expression of nEGFR in healthy oral mucosa, oral premalignant changes, and OSCCs. (**a**) Negative immunohistochemical expression of nEGFR in healthy oral mucosa. Magnification 200×. (**b**) Oral leukoplakia with moderate dysplasia and weak expression of nuclear EGFR (+). Magnification 400×. (**c**) Oral erythroplakia with moderate expression of nuclear EGFR (++). Magnification 200×. (**d**) Weak expression of nEGFR in well-differentiated OSCC (+). (**e**) Strong nuclear staining for nEGFR in more than 35% of cells in moderately differentiated OSCC (+++). Magnification 200×. (**f**) Strong immunohistochemical staining for nEGFR in the nucleus and moderate staining for EGFR in the cytoplasm of OSCC. Magnification 1000×.

In the control group, most samples (53/59) showed negative nEGFR expression (0), only 6 samples showed weaker expression of nEGFR (+), and moderate or strong expression (++/+++) was not found in any sample. Expression of mEGFR in the same group showed negative expression (0) in the largest number of samples (37/59), whereas weak expression of mEGFR (+) was present in 15 samples. Moderate expression of mEGFR (++) was detected in a smaller number of samples (7/59), while strong expression of mEGFR (+++) was not found in any sample (Figure 9, Figure 10 and Figure 11).

In the group of subjects with premalignant changes, a smaller number of samples (6/50) showed negative nEGFR (0), whereas the majority of subjects showed weak expression of nEGFR (+) (18/50) or moderate expression (++) (20/50). A smaller number of samples (6/50) showed strong expression of nEGFR (+++). Analysis of mEGFR revealed negative expression (0) in the majority of subjects (15/50), weak expression (+) in some subjects (11/50), and moderate expression (++) in a small number of subjects (4/50). Strong expression (+++) of mEGFR was detected in most samples (20/50) (Figure 9, Figure 10 and Figure 11).

The strongest expression of both nEGFR and mEGFR was observed in the group of subjects with OSCC; nEGFR was moderately to strongly expressed in 30/50 samples (++/+++), and 22 samples had negative or weak expression (0/+). In 33/52 samples, mEGFR was moderately to strongly expressed (++/+++), and in 19 samples, expression was weak or negative (0/+) (Figure 9, Figure 10 and Figure 11).

Comparisons of nEGFR and mEGFR expression between the groups of subjects analyzed showed statistical significance (χ^2^ = 85.96, *p* < 0.0001; χ^2^ = 70.40, *p* < 0.0001).

**Figure 12:**
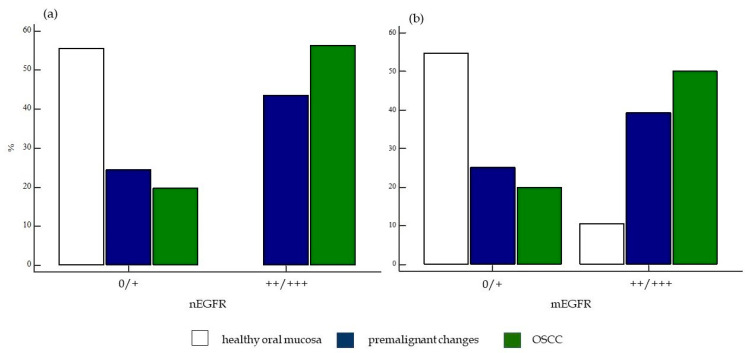
Presentation of the difference between weak and strong expression of nEGFR and mEGFR between the studied groups of subjects. Comparison of weak and strong expression of (**a**) nEGFR and (**b**) mEGFR between the analyzed groups of subjects revealed statistical significance (χ^2^ = 49.85, *p* < 0.0001; χ^2^ = 34.05, *p* < 0.0001). Legend: nEGFR—nuclear EGFR; mEGFR—membrane EGFR; 0—negative and weak expression, 1—moderate and strong expression.

When the frequency of nEGFR expression was compared between the studied groups, a statistically significant difference was found in the expression of nEGFR in the different groups. The frequency of moderate (++) and strong nEGFR expression (+++) was significantly higher in the group of patients with OSCC and premalignant changes than in the group of healthy subjects (χ^2^ = 49.85, *p* < 0.0001). A difference in the expression of mEGFR was also observed in the different patient groups, with a significantly higher frequency of moderate (++) and strong expression of mEGFR (+++) in the group of patients with OSCC and premalignant changes compared with the group of healthy controls. (χ^2^ = 34.05, *p* < 0.0001) (Figure 12).

#### 2.1.6. Correlation of nEGFR Expression with mEGFR and Markers of Cell Cycle, Cell Proliferation, and Tumor Stem Cells in Healthy Oral Mucosa, Premalignant Changes, and Invasive Oral Squamous Cell Carcinoma

In the group of healthy subjects, the correlation of expression between the tested markers did not show statistically significant results, which are therefore not shown.

In the group of patients with premalignant changes, a statistically significant positive correlation was observed between nEGFR and Ki-67 (ρ = 0.45, *p* = 0.001), p53 (ρ = 0.50, *p* = 0.0002), cyclin D1 (ρ = 0.42, *p* = 0.002), mEGFR (ρ = 0.54, *p* < 0.0001) and ABCG2 (ρ = 0.42, *p* = 0.002). A statistically significant correlation was observed between mEGFR and Ki-67 (ρ = 0.51, *p* = 0.0002), p53 (ρ = 0.50, *p* = 0.0002), nEGFR (ρ = 0.54, *p* < 0.0001), cyclin D1 (ρ = 0.38, *p* = 0.005), and ABCG2 (ρ = 0.49, *p* = 0.0003).

In the group of patients with premalignant changes, a statistically significant positive correlation between the degree of dysplasia and nEGFR (ρ = 0.60, *p* < 0.0001), Ki-67 (ρ = 0.42, *p* = 0.002), p53 (ρ = 0.50, *p* = 0.0002), cyclin D1 (ρ = 0.35, *p* = 0.01), mEGFR (ρ = 0.53, *p* = 0.0001) was also detected, while ABCG2 showed no significant correlation (ρ = 0.24, *p* = 0.10).

Considering the association of nEGFR with cell analyzed markers in the OSCC group, a statistically significant positive correlation was observed between nEGFR and Ki-67 (ρ = 0.31, *p* = 0.002), p53 (ρ = 0.30, *p* = 0.03), and mEGFR (ρ = 0.31, *p* = 0.02), while the correlation with cyclin D1 (ρ = 0.20, *p* = 0.16) and ABCG2 (ρ = 0.21, *p* = 0.12) was not observed. A statistically significant correlation was observed between mEGFR and Ki-67 (ρ = 0.34, *p* = 0.01), p53 (ρ = 0.37, *p* = 0.006), and nEGFR (ρ = 0.31, *p* = 0.02), while the correlation with cyclin D1 (ρ = 0.07, *p* = 0.62) and ABCG2 (ρ = −0.18, *p* = 0.19) was not observed.

#### 2.1.7. Association of Protein Expression of nEGFR and mEGFR and of Ki-67, p53, Cyclin D1, and ABCG2 with Clinicopathologic Parameters in Invasive Oral Squamous Cell Carcinoma

We analyzed the risk factors of alcohol and smoking, TNM stage of tumor, tumor localization, regional metastases, number of positive lymph nodes, histologic grade, lymphovascular invasion, perineural invasion (PNI), extranodal extension (ENE), margins, comorbidities, disease progression, HPV status, occurrence of another primary tumor, and death from the primary disease or another disease (Table 2). Statistical significance was found in the analysis of tumor sites in which expression of nEGFR and mEGFR was observed. No statistically significant difference was observed in the expression of nEGFR and mEGFR in relation to the other listed clinical data and pathohistological characteristics of the tumor. In the studied group of samples, the average tumor thickness was 7.5 ± 6.86 mm and the tumor size was 2.9 ± 1.39 cm.

When comparing the relationship between the expression of Ki-67 proliferation index and clinicopathological features of patients with OSCC, no statistical significance was found.

When comparing the relationship between the expression of p53 protein and the clinicopathologic features of patients with OSCC, PNI and death from underlying diseases or other diseases were statistically significant. Tumors without PNI had a significantly higher frequency of p53 protein expression than tumors with PNI (*p* = 0.02).

When comparing the association of cyclin D1 expression with clinicopathologic features of patients with OSCC, no statistical significance was found.

When comparing the association of ABCG2 expression with clinicopathologic features of patients with OSCC, statistical significance was found for PNI, whereas no statistically significant differences were found for other parameters.

### 2.2. Survival Analysis

Only patients with OSCC were included in the survival analysis, and follow-up data were available for all patients. Patients’ lifespan was followed from the time of diagnosis and/or surgery until last follow-up or death. All patients were treated surgically. The median follow-up time was 32.26 months with a range of 1 to 98 months. During the follow-up period, 18/52 patients died, of which 10 patients died from the underlying disease and 8 patients died from another cause of death and were censored in the analysis of experience. In addition, disease progression to a higher stage was observed in 12/52 patients during follow up. The median time to disease progression was 15 months with a range of 8 to 84 months.

For all biomarkers analyzed, the previously described cut-off values were used to divide patient groups into those with high or low expression of the tested proteins. In the analysis of survival, the influence of the parameters on overall survival was first determined by the Kaplan–Meier method, and the difference between survival curves was determined by the log-rank test.

**Figure 13:**
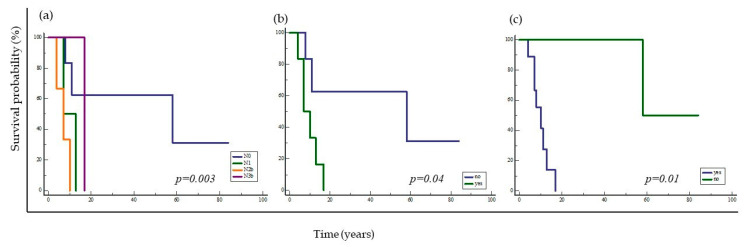
Kaplan–Meier survival curve considering the influence of clinicopathologic tumor characteristics in patients with OSCC. The curve shows significantly shorter survival of patients with higher N stage disease (*p* = 0.003) (**a**), lymphovascular invasion (*p* = 0.04) (**b**), and presence of a second primary tumor (*p* = 0.01) (**c**).

When analyzing the influence of tumor clinicopathologic characteristics on overall patient survival, only a difference in survival between patient groups was found with respect to regional metastases (*p* = 0.03), lymphovascular invasion (*p* = 0.04), and the presence of a second primary tumor (*p* = 0.01) (Figure 13). Other previously described clinical and pathological features of the tumor had no effect on overall patient survival. It is important to note that the margin of the preparation, which has been shown to have an impact on overall experience, was negative in all samples and therefore was not statistically significant in this study for monitoring patient experience.

**Figure 14:**
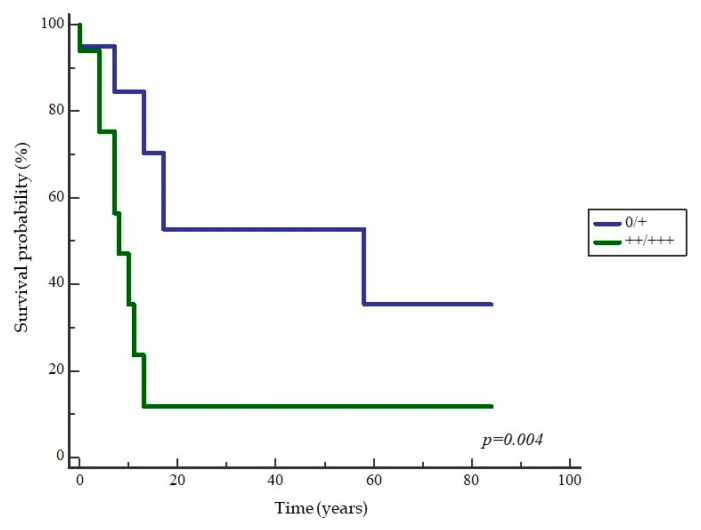
Kaplan–Meier survival curve in relation to nEGFR expression in patients with OSCC. The curve shows significantly shorter survival of patients with moderate and strong expression of nEGFR in tumor tissue (*p* = 0.004).

In addition, analysis of patients’ overall survival based on the analyzed proteins showed a statistically significant association between nEGFR and survival (*p* = 0.004). Patients with moderate and strong expression of nEGFR (++/+++) in tumor tissue had significantly shorter overall survival compared to patients with negative and weak nEGFR (0/+) (Figure 14). This analysis revealed no difference in survival between patient groups with respect to expression of mEGFR, Ki-67, p53, cyclin D1, and ABCG2.

## 3. Discussion

Oral squamous cell carcinoma (OSCC) is the most common malignant tumor of the head and neck (HNSCC), i.e., the sixteenth most common cancer worldwide, with a relatively poor five-year survival rate of approximately 55%, despite significant advances in diagnostic and therapeutic procedures over the past 30 years [2,17]. Surgical resection of the tumor with or without neck dissection remains the method of choice in the treatment of OSCC. Adjuvant radiotherapy or chemoradiotherapy is performed depending on the pathohistological features of the tumor [1]. Although the presence of dysplasia in oral leukoplakia and oral erythroplakia is the most important prognostic factor for malignant transformation, the available diagnostic classifications of dysplasia have numerous shortcomings. One of the main reasons is the subjectivity of the observer and the resulting poor reproducibility of the diagnostic criteria, which has been confirmed by numerous studies showing a weak correlation between the degree of dysplasia and the malignant transformation of potentially malignant oral disorders (OPMD) [18,19,20,21]. Consequently, new biomarkers need to be found that can be used in routine practice to assess the risk of malignant transformation from premalignant changes in OSCC. Late detection of OSCC, the occurrence of locoregional disease recurrence, and metastatic disease are characterized by poor prognosis, and there is a need for the development of biomarkers for early detection of disease, more reliable prediction of disease prognosis, and selection of appropriate therapy [20]. The fact that patients with similar clinicopathologic features often have different disease progression, response to therapy, and treatment outcome points to the need to identify novel prognostic factors that more accurately determine the biologic behavior of tumors. Biomarkers of genomic instability could accurately measure the risk of malignant transformation from premalignant changes in OSCC and the risk of spread and metastasis of the primary tumor to regional lymph nodes and distant organs [19,22]. According to the results of this study, the mean age of patients with OSCC was 55.21 years, and the cancer occurred twice as often in men (67.3%; 35/52). The mean age of patients with premalignant changes was 64.22 years, and women were slightly more frequently affected (54%; 27/50). The distribution of age and sex in patients with OSCC depends on geographic location, and our data are consistent with those of European countries [23,24]. The most frequent localizations of premalignant changes and OSCC in the oral cavity were the tongue and the floor of the oral cavity (75%, 39/52 and 64%, and 32/50, respectively), which is consistent with the literature. The aforementioned areas have been shown to be predilection sites for premalignant changes and OSCC due to the deleterious effects of carcinogens that accumulate in the so-called salivary pool. For head and neck tumors, numerous diagnostic and prognostic markers have been investigated in clinical studies, but their clinical significance remains questionable [5,22]. Recent discoveries related to a completely new way of regulating cell proliferation and apoptosis through the independent action of EGFR in the nucleus of numerous tumors, such as ovarian, breast, oropharyngeal, laryngeal, and esophageal carcinoma, have been the basis for studying premalignant and malignant changes in the oral cavity, where the role of this receptor had not been previously elucidated [8,11,12,13,14,15,16]. In addition to nEGFR, we also analyzed the expression of markers of cell cycle and proliferation (Ki-67, cyclin D1, p53, mEGFR) and markers of tumor stem cells (ABCG2) involved in oral carcinogenesis. Ki-67 is considered one of the most important immunohistochemical markers of cell proliferation and aggressiveness of numerous tumors, such as breast, lung, prostate, cervical, soft tissue, and central nervous system tumors, and its excessive expression is a poor prognostic sign [25,26,27]. Although the results of studies on Ki-67 and HNSCC are conflicting, there are a larger number of studies indicating that overexpression of Ki-67 is associated with progression of OPMD and with a higher rate of locoregional recurrence as well as distant metastasis and worse OS, DFS, RFS, and MFS in patients with OSCC [28,29,30,31]. Moreover, expression in OSCC was inversely proportional to tumor differentiation. A statistically significant difference in the expression of Ki-67 was demonstrated between the groups of patients with OSCC on the one hand and subjects with premalignant changes and the control group on the other hand. In our study, the percentage proliferation index was significantly higher in the group of cancer patients compared with the healthy subjects and those with premalignant changes, whereas no statistically significant difference was demonstrated between the control group and the subjects with leukoplakia and erythroplakia. In the subjects with premalignant changes, the expression of Ki-67 increased statistically significantly with the progression of dysplasia (*p* = 0.005), which is consistent with data from the literature. Sharma, like us, demonstrated a positive correlation between Ki-67 expression and disease progression from low-grade dysplasia to high-grade dysplasia to OSCC in 65 subjects, 40 of whom had OSCC and 25 of whom had premalignant changes [32]. The increase in Ki-67 expression with progression of dysplasia in leukoplakias is the result of an observational study conducted by researchers from India in 2020 on 786 subjects with leukoplakia, of whom 126 had epithelial dysplasia, and 14 patients developed OSCC [33]. Similar results were also obtained by Dwivedi et al. [34]. Comparison of Ki-67 expression with clinicopathologic features of patients with OSCC did not reveal statistical significance. Birajdar’s studies found increased expression of Ki-67 in poorly differentiated carcinomas compared with well-differentiated OSCC [35]. In our subject sample, we did not demonstrate a statistically significant association between Ki-67 expression and histologic differentiation of OSCC. The p53 protein is classified as a tumor suppressor protein, and due to its multiple roles in cellular homeostasis, it is classified as a central regulator of the genome. More than 50% of malignancies exhibit excessive p53 expression caused by p53 gene mutations and epigenetic alterations [36,37]. Numerous genetic analyzes have shown a high frequency of p53 gene mutations in the early stages of carcinogenesis in HNSCC (more than 70% of tumors) [38]. In our study, we found significantly higher expression in the group of subjects with OSCC and subjects with premalignant changes compared with the control group, and a statistically significant difference was also found between all analyzed groups. Considering the significant increase in p53 expression in premalignant changes compared with healthy mucosa and the evidence that expression correlates with malignant transformation of OPMD, as well as the small number of influences of p53 protein on patient experience, it is reasonable to assume that inactivation of this protein is crucial in the early phase of oral carcinogenesis. In our studies, the trend of increased expression of p53 is observed in advanced cancers compared with the early stages of the disease. A statistically significant association between p53 protein expression and clinicopathologic features of patients with OSCC was demonstrated for PNI and death from underlying disease or other diseases. OSCC without PNI had a significantly higher frequency of p53 expression than tumors without PNI (*p* = 0.02). The origin of PNI in head and neck tumors is still largely unknown due to the distinct molecular complexity of the process. It is known that the presence of PNI in HNSCC is a negative prognostic sign, and it is recommended that patients with OSSC and PNI receive postoperative adjuvant radiotherapy. The lack of studies investigating the impact of mutation and overexpression of p53 protein on the occurrence of PNI in patients with OSCC speaks to the complexity of the mechanism of nerve invasion itself. One of the signaling receptors on tumor cells associated with cell migration and PNI is Galanin receptors 2 (GALR2), which is thought to play a very important role in regulating PNI in HNSCC. Banerjee et al. induced cell lines from HNSCC to overexpress GALR2 and observed that this stimulated cell proliferation and tumor cell survival via activation of ERK and Akt in vitro and cell proliferation in vivo [39]. Thus, he proved that GALR2 receptor overexpression plays a protumoral role in HNSCC cells, whereas Kanazawa observed the opposite effect of GALR2 in patients with HNSCC and overexpression of p53 mutations [40]. According to our results, PNI occurred more frequently in advanced disease when the expression of p53 protein was also reduced, suggesting that the effect of p53 expression on the development of PNI is inversely proportional, and that p53 plays a much more important role in early carcinogenesis. Furthermore, the impact of p53 protein overexpression on overall survival of patients with OSCC is unknown. Khan failed to demonstrate a statistically significant correlation with clinicopathologic parameters in a sample of 29 OSCC [41]. In a prospective study by Ogmundsdóttir and colleagues on a sample of 144 subjects with premalignant (OL and lichen ruber planus) and malignant changes of the oral mucosa, they concluded that p53 gene mutations can persist in benign lesions of the oral mucosa for many years without developing malignant disease. Moreover, no association was found between p53 protein expression and OSCC recurrence or disease-related survival, whereas overall survival was shortened in patients overexpressing this protein [42]. Cyclin D1 regulates the cell cycle and plays an important role in tumorigenesis of numerous tumors, including OSCC. Cyclin D1 overexpression has been found in 32 to 88% of malignant tumors [43,44,45]. According to the results of numerous studies, cyclin D1 is considered a negative independent prognostic factor and biomarker for the aggressiveness of OSCC [46]. Huang demonstrated in 264 subjects with OSCC that overexpression of cyclin D1 was associated with higher tumor stage and poorly differentiated carcinomas, higher rate of regional metastases, and worse DFS and OS (282). In our study, we followed the dynamics of increased expression of cyclin D1 from normal mucosa to premalignant changes to OSCC demonstrating strong expression in 82.6% of tumors (43/52). Moharii et al. observed something similar in 75 patients with premalignant and malignant changes in the oral cavity [47]. We found no statistically significant difference in the dysplasia group in subjects with premalignant changes. When comparing the relationship between cyclin D1 expression and clinicopathologic features in patients with OSCC, no statistically significant differences were found, and there was no effect on the overall outcome. Numerous studies on OSCC have demonstrated the association between cyclin D1 expression and clinicopathologic and prognostic factors in patients with OSCC. Carlos de Vi-cente, Das, Gupta, and Guimaraes found higher expression of cyclin D1 in higher T-stage tumors, which was also confirmed by Zhao 2014 in his meta-analysis [48,49,50,51]. Wang and Liu found a statistically significant correlation between cyclin D1 expression and tumor thickness and depth of invasion (DOI) [52]. Many authors have demonstrated the increased expression of cyclin D1 in premalignant transformation and the positive dynamics of increased expression with the progression of dysplasia and progression to OSCC and disease progression. Numerous studies have also demonstrated the association between cyclin D1 expression and disease stage N, which was also confirmed by two meta-analyzes in 2014 and 2015 [53,54]. Interestingly, numerous authors such as Bov, Miyamoto, Lam, and Huang have found an increase in cyclin D1 expression with a decrease in tumor differentiation, i.e., an increase in the histological grade of the tumor [55,56]. The results of the present study suggest the opposite: the higher the histologic grade, the lower the expression of cyclin D1. Saawarn showed an increase in cyclin D1 expression with OSCC differentiation in 40 subjects, which is consistent with our observations [56]. Similar results were obtained by Angadi, Krishnapillai, and Das [57,58]. The relationship between cyclin D1 and the degree of tumor differentiation is controversial and has not yet been clarified. This discrepancy in results is partly explained by the use of different histologic criteria for determining cyclin D1 expression. Another explanation was provided by Woods and colleagues in a study of oral keratinocyte cell lines in which stimulation of cyclin D1 expression increased cell proliferation but did not block cell differentiation [59]. This suggests that cyclin D1 is able to directly affect transcriptional regulation of genes involved in oral keratinocyte differentiation independently of CDK. Therefore, Ohnishi concluded in 2014 that cyclin D1 is involved not only in cell proliferation but also in cell differentiation and prevention of cell death in OSCC [60]. Further studies are needed to investigate in detail the role of cyclin D1 in oral keratinocyte differentiation and whether it can modulate cell differentiation in OSCC toward less aggressive histological stages with better prognosis. Expression of the ABCG2 protein, also known as Breast Cancer Resistance Protein (BCRP), was recently discovered as a potential biomarker for the severity of OPMD and OSCC [61,62,63]. It is responsible for resistance to numerous drugs in many tumors and is one of the markers of tumor stem cells [64,65]. ABCG2 is overexpressed in the side population of tumor stem cells, which play an important role in oral carcinogenesis [66]. When we analyzed the expression of ABCG2, we found a statistically significant difference between the studied groups. The weakest expression of ABCG2 was detected in control mucosa, with an increase in immunoreactivity in the group of patients with premalignant changes and the highest expression of the protein in subjects with OSCC. We also demonstrated a significant increase in ABCG2 expression with progression of dysplasia in premalignant changes. A study by Shi et al. demonstrated the association between ABCG2 expression in oral lichen ruber planus and an increased risk of malignant transformation in a sample of 110 patients, whereas Feng confirmed the potential of ABCG2 in predicting malignant transformation by analyzing ABCG2 expression in healthy oral mucosa, premalignant changes, and oral cavity cancer in 8 cell lines and 189 subjects [62,63]. A detailed analysis of the sublocalization of ABCG2 immunoreactivity has not been described, although several papers mention the possible importance of intracellular localization of the protein. Several studies have observed membranous and nuclear expression of ABCG2 in malignant tumor cells, such as lung and laryngeal carcinomas and glioblastoma multiforme [67,68,69]. A possible novel role of ABCG2 within the nucleus as a transcriptional regulator involved in modulation of metastasis has been proposed in lung cancer [68]. In our samples, we observed immunoreactivity in the nucleus in addition to membrane and cytoplasmic expression of ABCG2. The main reason for the positive ABCG2 immunoreactivity in the different sublocalizations remains to be clarified in future studies. Analysis of the association between ABCG2 expression and clinicopathological features of OSCC revealed a statistically significant association with PNI (*p* = 0.02), while no statistically significant differences were found for the other parameters analyzed. The role of ABCG2 in OSCC is not known, and there are few studies in the available literature that have analyzed this role, mainly due to the resistance of OSCC to chemotherapy, following the findings related to breast cancer. Yanamoto et al. demonstrated that overexpression of ABCG2 in OSCC was associated with PNI, a higher rate of regional metastasis, and local recurrence in 89 subjects [70]. 

The concept of concomitant chemoradiotherapy, which includes the use of postoperative radiotherapy and cisplatin-based chemotherapy, has remained unchanged since its introduction in the 1960s [71]. Although a positive effect on locoregional disease control and survival has been demonstrated in patients with HNSCC, 5-year overall survival has not been significantly prolonged in advanced tumors and ranges from 30% to 60% [72]. The discovery of EGFR overexpression in numerous malignancies and its oncogenic effect on gene expression, cell proliferation, angiogenesis, apoptosis, cell motility and adhesion, and metastasis has led to the development of numerous drugs that inhibit its action. Given the overexpression of EGFR in more than 90% of head and neck tumors and the poorer survival of these patients, it was hypothesized that patients would benefit greatly from the use of anti-EGFR drugs [72]. Numerous inhibitors have been developed. The best known is cetuximab, a chimeric IgG1 monoclonal antibody that binds to the extracellular domain of the EGFR membrane and is approved in combination with radiotherapy for the treatment of advanced HNSCC and as monotherapy for locoregional recurrence and metastatic disease. In 2011, the FDA approved the use of cetuximab in combination with cisplatin-based chemotherapy and 5-FU to treat locoregional recurrence and metastatic disease. However, the fact that less than 20% of HNSCC respond to cetuximab and that concomitant use with chemoradiotherapy does not significantly improve disease outcomes in advanced disease is quite discouraging. Intensive work is being performed to identify possible causes of resistance to cetuximab in tumors with high EGFR expression [73,74]. One of the possible explanations for resistance is translocation of the receptor into the nucleus, which can be induced by irradiation, cetuximab, the effects of cisplatin, increased expression of EGFR ligands, and activation of the src kinase family [75]. This suggests that EGFR in the nucleus may influence the expression and transcription of numerous genes involved in tumorigenesis via other, as yet unknown, multiple downstream signaling pathways. Moreover, in addition to cetuximab, drugs have been developed that inhibit tyrosine kinase activity by binding to the intracellular domain of EGFR. Tyrosine kinase inhibitors (TKIs) such as gefitinib have shown limited clinical efficacy, responding in only 10% to 15% of patients with HNSCC. Less than 5% of HNSCC have EGFR mutations, which may partially explain the reported tumor resistance to TKIs [75]. Recent studies began to focus attention on the cellular sublocalization of EGFR, and it was found that this receptor can be overexpressed in the cytoplasm (cEGFR) as well as in the nucleus (nEGFR) in addition to the membrane, with potentially novel implications for the expression of numerous genes. These results indicate that there are still many unknowns in the action of EGFR that need to be investigated. There are few papers in the literature that have investigated the effects of cEGFR and nEGFR expression in HNSCC, and no single study focused on OSCC [16]. According to the available literature, this study is the first to investigate the expression and impact of nEGFR in premalignant and malignant changes of the oral cavity on malignant transformation and disease progression. A large number of studies have investigated the significance of EGFR overexpression by immunohistochemical methods in HNSCC, which represent a very large heterogeneous group of tumors with different biological behaviors [11,12,13,14,15,16,76]. Results are often contradictory, in part because of inconsistent quantification of immunohistochemical receptor expression, neglect of receptor expression in single cell compartments, and inclusion of different head and neck tumors in the studies.

Our results show a statistically significant difference in the expression of mEGFR and nEGFR between the studied groups (*p* < 0.0001) with an increase in moderate and strong expression and with the progression of genetic instabilities from the healthy control group, and premalignant changes to the OSCC. The results of this study regarding membrane expression of EGFR in premalignant and malignant changes are consistent with the available results from the literature. Mirza et al. found overexpression of mEGFR in 129 subjects in 51% of patients with premalignant changes and in 67% of patients with OSCC. Furthermore, they demonstrated that overexpression of mEGFR in patients with OSCC negatively affected 5-year OS and was associated with a higher risk of disease recurrence [77]. In 2018, Singala examined the expression of several molecular markers (EGFR, p53, c-erbB2) in 40 oral leukoplakias and 40 OSCC and also found a significant increase in EGFR expression with progression of premalignant changes in OSCC. They concluded that excessive co-expression of p53 and EGFR may indicate a higher risk of malignant transformation from leukoplakia to OSCC [78]. Ries reached similar conclusions when studying the malignant transformation of 98 leukoplakias, particularly emphasizing that expression of EGFR correlated more strongly with malignant transformation in relation to the degree of dysplasia [79]. Thus, the results of most studies on premalignant and malignant transformation of the oral cavity are consistent with the results of this study when we talk about the expression of mEGFR. In the available literature, there is no single study that investigated the expression of nuclear EGFR in premalignant and malignant transformation of the oral cavity, and therefore we cannot compare our results with the literature. A significant increase in the expression of both membrane and nuclear EGFR already in premalignant changes compared with the control group suggests that these two proteins play an important role in early oral carcinogenesis. When analyzing the correlation of nEGFR expression with mEGFR and markers of the cell cycle, cell proliferation and tumor stem cells in the studied groups, interesting results were found. In the group of patients with premalignant changes, a statistically significant positive correlation was observed between nEGFR and Ki-67, p53, cyclin D1, mEGFR, and ABCG2. Analysis of the correlation between the degree of dysplasia and the markers studied showed a statistically positive correlation with an increase in the degree of dysplasia and an increase in the expression of nEGFR, Ki-67, p53, cyclin D1, and mEGFR, whereas ABCG2, although not statistically significant, showed a visible positive trend. Similar observations of correlation between the studied cell cycle markers and tumor stem cells were demonstrated in patients with OSCC. A statistically significant positive correlation was observed between nEGFR and Ki67, p53, and mEGFR, whereas the correlation with cyclin D1 and ABCG2 was not observed but a positive trend was evident. A statistically significant correlation was observed between mEGFR and Ki67, p53, and nEGFR, whereas the correlation with cyclin D1 showed only a positive statistical trend. Cancer progression occurred in 12 patients with OSCC (23.1%), and 10 patients (19.2%) died as a result of OSCC. The correlation of the analyzed markers was not related to disease progression or death from OSCC. The above results of correlation of nEGFR with other markers studied cannot be compared with data from the literature because of the lack of studies that have investigated nEGFR in premalignant and malignant changes of the oral cavity. We can discuss the above results in the context of studies on other malignancies of the head and neck. Positive correlations between nEGFR and other investigated biomarkers in premalignant changes and dysplasias can be explained by the influence of EGFR on stimulating cell proliferation and blocking apoptosis, which has been confirmed in previous studies [7,8,9,10,11]. It is known that EGFR in the nucleus can activate transcription of cyclin D1 by binding to the promoter site of the CCND1 gene, which may explain the positive correlation between the aforementioned biomarkers. Blocking apoptosis is also possible by reducing CKI activity caused by mutations and overexpression of EGFR [7]. Ki-67 expression is closely related to cell proliferation and tumor cell growth, which is consistent with our results and the fact that an increase in Ki-67 expression is expected with the progression of dysplasia and OSCC. This was demonstrated by Jing et al. when they analyzed 396 samples of OSCC, oral dysplasia, and healthy oral mucosa [28]. Numerous studies have confirmed the high expression of the p53 gene in OSCC (54%, 75%, 95%, and 65%), and a trend toward increased expression with progression of premalignant changes in the oral cavity from hyperplasia to dysplasia to cancer has been noted [80,81,82]. Disruptive and nondisruptive mutations of the p53 protein result in impaired function of this protein with the inability to induce apoptosis in damaged cells. Liu demonstrated in hepatocellular carcinoma cell lines that nEGFR can affect cell apoptosis by stimulating the expression of SOS1, which then activates the HRAS/PI3K/AKT pathway, leading to nuclear translocation of p-AKT and Bcl-2. The interaction between p-AKT and ASPP2 facilitates the binding of BcL-2 to p53, leading to the release of p53 from the pro-apoptotic gene promoter. Activation of the HRAS/PI3K/AKT pathway by nEGFR-induced SOS1 also inhibits cisplatin-induced apoptosis [83]. In 2021, Marijić et al. examined the expression of mEGFR and nEGFR in laryngeal polyps, dysplasias, and squamous cell carcinomas, and confirmed a significantly higher frequency of strong nEGFR expression in cancer, dysplasias, and polyps, as well as strong expression of mEGFR in cancer and laryngeal dysplasias compared with polyps [16]. This was confirmed by our studies on premalignant changes and OSCC. In the group of subjects with OSSC, we observed a positive correlation of membrane and nuclear EGFR expression in agreement with the results of Psyrri et al. in oropharyngeal carcinomas [14]. Marijić demonstrated the inverse expression of mEGFR and nEGFR in squamous cell carcinomas of the larynx and concluded that only one EGFR signaling pathway, membrane or nuclear, controls further carcinogenesis in tumors [16]. The results of this study suggest that both EGFR signaling pathways influence carcinogenesis, possibly stimulating each other and possibly acting independently. One of the aims of this study was to analyze the expression level of nEGFR in relation to the studied clinical and pathological features of patients with OSCC. We did not find a single statistically significant association, which is similar to the results of Marijić and Psyria, on laryngeal and oral cavity cancer, whereas there are no comparable studies on the association between nEGFR and OSCC in the available literature [14,16]. When analyzing mEGFR in relation to the investigated clinicopathologic features of OSCC, we also did not find a single statistically significant correlation. Shahsavari failed to demonstrate any correlation between mEGFR expression and clinicopathologic features of OSCC, consistent with our findings [84]. In contrast, Costa et al. demonstrated the negative impact of EGFR on disease progression in individuals younger than 40 years, which contradicts our observations [85]. Abbas demonstrated an increase in mEGFR expression with an increasing histologic grade of the tumor in 30 OSCC and concluded that EGFR can be used as an indicator of tumor aggressiveness [86]. All of the aforementioned studies were performed on a small number of subjects, and there is a need for large multicenter studies that demonstrate the true relationship between membrane and nuclear EGFR expression and tumor clinicopathologic features.

In patients with OSSC, we additionally analyzed the impact of the investigated biomarkers and tumor clinicopathologic features on the overall patient experience. The median follow-up time of patients was 32.26 months. During this time, 18 patients died, ten of them from oral cavity cancer and the other eight from another cause unrelated to OSCC. Nine patients developed a second primary tumor during the follow-up period. Patients with OSCC who had regional disease, lymphatic invasion and the presence of a second primary tumor had significantly worse overall survival compared with patients without these features. According to the results of Brand’s study of 594 patients with OSCC, the 1-year, 5-year, and 10-year cumulative risks of other primary tumors and disease recurrence were 17%, 30%, and 37%, respectively, and almost all locoregional disease recurrences occurred within the first 2 years after treatment. Other primary tumors significantly worsen the overall patient experience, making lifelong surveillance of patients with head and neck tumors extremely important because of the possible occurrence of other tumors in the oral cavity, which is genetically damaged by the accumulation of known risk factors. The lung and liver are the most common sites for other primary tumors outside the head and neck region, and it is occasionally necessary to screen with radiologic methods [87]. The presence of metastases in the regional lymph nodes decreases the survival rate of oral cavity cancer by 50% for each individual stage of disease. According to the TNM classification, the N stage of the disease is divided into four categories (N0-N3). The higher the N stage of the disease, the shorter the overall survival. In the presence of regional metastases, the patient is at least in the III stage of disease with a significantly reduced 5-year survival rate of about 51% compared with localized disease (stage I/ II), in which the survival rate is about 82% (1–2, 18). A Kaplan–Meier analysis of survival of patients with OSCC, depending on the expression of the markers studied, revealed a statistically significant shorter overall survival in patients with moderate and strong expression of nEGFR in tumor tissue compared to patients with weak expression. According to the available literature, these are the first results of a study investigating the impact of nEGFR expression in OSCC on overall patient survival (OS). Marijić demonstrated the negative impact of excessive expression of nEGFR on overall survival in laryngeal carcinomas, whereas Psyrri proved the same in oropharyngeal tumors [14,16]. Schmidt-Ullrich et al. demonstrated that irradiation of tumors leads to activation and internalization of EGFR in the nucleus [88]. Dittman demonstrated that EGFR and DNA-PK form a complex in the nucleus after irradiation, leading to increased DNA repair activity and acquired resistance to radiotherapy [89]. Treatment of carcinomas with cisplatin has also been shown to induce nuclear translocation of EGFR and increase resistance to chemotherapy [75]. This suggests that nEGFR plays an important role in DNA damage repair, which may explain the results of this study. In addition, we performed prognostic analyzes of clinicopathologic parameters for disease progression and death in OSCC. Alcohol consumption, clinical tumor stage, and PNI were found to be strong predictors of disease progression, whereas the presence of regional metastases, PNI, the number of positive lymph nodes, LVI, clinical tumor stage, and alcohol consumption were found to be strong predictors of death in patients with OSCC. The above observations are consistent with data from the literature [1,4,90].

Finally, the role of nEGFR in malignant tumors of the head and neck has not been adequately studied, whereas its role in premalignant and malignant changes in the oral cavity is unknown according to the available literature. The rapid increase in research related to the nuclear expression of EGFR was triggered by discoveries about the effects of this receptor on resistance to chemotherapy and radiotherapy. This demonstrates the complexity and inadequate knowledge of the signaling pathways mediated by EGFR. According to the available literature, this is the first study to investigate the impact of nEGFR expression in premalignant and malignant changes of the oral cavity and the negative impact on the overall experience of patients with OSCC. The above results suggest that nEGFR plays an important role in the development of OSCC. With readily available and convenient immunohistochemical methods, we can determine the expression of this receptor in the nucleus and widely apply it in clinical practice to more accurately determine the malignancy risk of precancerous lesions of the oral cavity compared with previous semiquantitative methods for determining dysplasia. Molecular quantification of the progression of premalignant changes in the oral mucosa would influence the type and extent of treatment and the frequency of patient follow-up. In OSCC resection, the application of molecular diagnostics could greatly alter the principles of tumor treatment by determining not only surgically or pathohistologically healthy margins but also the need for elective neck dissection or adjuvant treatment. Further studies in a large sample of subjects are needed to additionally and comprehensively investigate the role of nEGFR in OSCC and its interaction with membrane and cytoplasmic epidermal growth factor receptors. The only drawback we would cite to the use of nEGFR is the somewhat weaker visualization of the immunohistochemical response, as it is still an experimental antibody where the experience of the pathologist in reading is very important.

## 4. Materials and Methods

### 4.1. Patients

The study involved 161 patients treated at the Department of Maxillofacial Surgery, Dubrava University Hospital. They were divided into three groups: 50 patients with premalignant changes (leukoplakia and erythroplakia), 52 patients with invasive oral squamous cell carcinoma, and 59 subjects in the control group who had their mucosa removed due to non-tumour disease. All patients were followed for a period of at least 5 years. Inclusion criteria for patients were: clinically and pathohistologically verified premalignant change or OSCC; primary surgically-treated patients with OSCC; available pathohistological material for immunohistochemical analysis; available clinically and pathohistologically relevant data from medical history, hospital information system, clinical oncology database, and cancer registry of the Croatian Institute of Public Health. Patients previously treated for head and neck malignancy, patients with insufficient samples for immunohistochemical analysis, and patients with inadequate follow up or incomplete medical documentation were not included in this study.

### 4.2. Pathohistological Samples

Paraffin-embedded archival specimens from biopsies of premalignant changes (leukoplakia and erythroplakia), resected primary OSCC, and excised oral mucosal tissues with nonmalignant disease were used for this study. To confirm the diagnosis and to determine the adequacy of the quality and quantity of the pathohistological material, two pathologists from the Department of Pathology and Cytology of Dubrava University Hospital examined the subjects’ specimens again separately. The specimens were first fixed in 10% buffered formalin (Kemika, Zagreb, Croatia), embedded in paraffin, cut into 3 to 4 µm thick sections, deparaffinized, and stained with hemalaun-eosin (HE).

### 4.3. Immunohistochemical Staining

In this study, 2–3 µm thick sections were prepared from the paraffin blocks and then dewaxed in a thermostat. To determine the expression of p53 and mEGFR proteins in the samples after deparaffinization, predigestion was performed in a thermobath (PT-link, DAKO, Glostrup, Denmark), followed by the use of “EnVision target Retrieval solution, High pH” (DAKO, Denmark), i.e., predigestion with exposure of epitopes by heat in a microwave oven with pH6 buffer to determine the expression of nEGFR and ABCG2 proteins. Immunohistochemical staining was performed using an automated immunohistochemical system (DAKO autostainer, DAKO, Denmark). For immunohistochemical staining, a “ready-to-use” p53 antibody (mouse monoclonal antibody, clone DO-7, DAKO, Denmark) was used with a 45-min incubation; an NCL-L EGFR antibody (Leica; Novocastra, Newcastle upon Tyne, UK) at dilution 1:50 with a 60-min incubation; an EGFR antibody (Thermo Fisher Scientific, Invitrogen, LSG Bioproduction, Waltham, M, USA), clone EGFR-1, at a dilution of 1:25 with a 90-min incubation; or ABCG2 antibody, clone B-1, at a dilution of 1:25 with a 90-min incubation. Immunohistochemical staining expression was detected by an indirect method using the EnVision detection kit (DAKO, Denmark). Subsequently, preparations were contrasted with hemalaun (1 min) and placed in an ascending series of alcohol (70–100%), and then in xylene and glass coverslip. Colon tissue served as a positive control for p53 and placental tissue for mEGFR, while paraffin-embedded breast tissue was used for nEGFR and ABCG2 according to the recommendations of the manufacturer of the antibodies tested. 

For immunohistochemical analysis of cyclin D1 and Ki-67 expression, pre-digestion was performed in the Ventana BenchMark Ultra instrument (Roche Diagnostics, Basel, Switzerland) with thermostats and ULTRA Cell Conditioning Solution after deparaffinization. Immunohistochemical staining was performed using an automated immunohistochemical system. The optiViewUniversal DAB detection kit (Ventana Medical Systems) was used for visualization. Cyclin D1 antibody (rabbit monoclonal antibody, clone EP12, DAKO, Denmark) at a dilution of 1:75 with an incubation time of 12 min at a temperature of 37 °C and Ki67 antibody (mouse monoclonal antibody, clone MIB-1, DAKO, Denmark) at a dilution of 1:75 with an incubation time of 16 min at a temperature of 37 °C were both used for immunohistochemical staining. The resulting complex was visualized with hydrogen peroxide and the chromogen DAB, which forms a brown precipitate visible under the light microscope. This was followed by contrasting with hemalaun (1 min) and running through an ascending series of alcohol (70–100%), xylene, and coverslip. Paraffin-embedded tonsil tissue was used as a positive control for cyclin D1 and Ki-67.

### 4.4. Evaluation of Immunohistochemical Staining

In assessing cell proliferation index (Ki-67) expression, we relied on numerous papers in the literature that set the “cut-off” value at 30% of positively-stained nuclei. We classified lesions with more than 30% positive nuclei as highly proliferative, whereas lesions with less than 30% positive nuclei were classified as weakly to moderately proliferative [91].

Immunohistochemical expression of p53 and cyclin D1 was based on the Allred scoring system combining staining intensity and percentage of positively-stained nuclei [92]. Depending on the percentage of positively-stained nuclei, we divided expression into five categories. We labeled the lesions that did not have a single positively-stained nucleus with number 0 (negative lesions), the percentage of positive nuclei up to 1% with number 1, the percentage of positive nuclei from 1–10% with number 2, the percentage of positive nuclei from 10–33% with number 3, the percentage of positive nuclei from 34–66% with number 4, and number 5 if the lesions had more than 67% positively-stained nuclei. We also divided the intensity of staining into three categories, so that we assigned the number 0 as negative intensity of staining for lesions, in which not a single nucleus was stained under high magnification on the light microscope (×400); the number 1 was assigned for lesions with weak intensity of staining, where the staining is visible only at high magnification (×400); number 2 was assigned for lesions with moderate staining intensity, where the colored lesions are easily visible even at low magnification (×100); and number 3 or strong staining was assigned for lesions where the staining is clearly visible at low magnification. We divided the total sum of values for intensity of nuclear staining (0–3) and the percentage of positively-stained nuclei (0–5) of lesions into three groups: (0)—negative lesions or lesions with weak expression (sum 0–2); (+)—lesions with moderate growth (sum 3–5); and (++)—lesions with strong expression (sum 6–8). To evaluate the immunoreactivity of the ABCG2 protein with an experimental antibody, we used a scoring system previously described by Abdulmajeed [93]. This classification system combined the intensity of staining (0 = no staining to 4 = dark brown staining) and the percentage of positively-stained epithelial cells (0% = score 0; <25% = score 1; 25–49% = score 2; 50–74% = score 3; 75–100% = score 4), and lesions were classified into four groups: (0)—negative lesions; (+)—lesions with weak expression (sum 1–2); (++)—lesions with moderate expression (sum 3–5); and (+++)—lesions with strong expression (sum 6–8). We assessed membrane expression of EGFR according to the work of Cho EY et al.: (0)—no membrane staining or positivity in ≤10% of cells; (+) incomplete membrane staining in >10% of cells; (++) weak to moderately complete membrane staining in >10% of cells; and (+++) strong and complete membrane staining in >10% of cells [17]. To quantify nEGFR expression, we used the criteria described by Lo et al. We divided nEGFR immunoreactivity into four groups depending on the percentage of positive cells: (0) no nuclear staining; (+) 1–17% cells with positive nuclear staining; (++) 18–35% of cells with positive nuclear staining; and (+++) > 35% of cells with positive nuclear staining [8].

### 4.5. Statistical Analysis

Statistical processing of the data was performed with the statistical computer program MedCalc, version 12.5.0 (MedCalc Software, Ostend, Belgium; https://www.medcalc.org, accessed on 15 May 2021), and the results were presented in tables and graphs. Values of continuous variables are presented as mean ± standard deviation. Categorical (qualitative) data are presented in frequencies and percentages. Analysis of the distribution of the measured variables (Kolmogorov–Smirnov test) determines the difference in the distribution of each variable; the normality of the distribution varies from parameter to parameter, so the one-way ANOVA test (for data with normal distribution) and the nonparametric Kruskal–Wallis method were used to compare more than two groups of subjects. A Student–Newman–Keuls post hoc test was used to test for differences between groups. In addition, the nonparametric Mann–Whitney test was used. Associations (correlations) between individual parameters were examined using the Pearson test or the Spearman test and the regression model, depending on the normality of the data distribution. To test for differences in nominal variables, Fisher’s exact test or the χ^2^ test was used. In addition, the odds ratio with the confidence interval was calculated for each variable. The relationship between the expression of the analyzed biomarkers and the overall survival of the subjects was assessed by the Kaplan–Meier method, and the difference between the survival curves was determined by the log-rank test. The potential prognostic value of the analyzed biomarkers was determined with the ROC (Receiver Operating Characteristic) analysis. Test results were considered significant when *p* ≤ 0.05.

## 5. Conclusions

The results of this study suggest a possibly important independent role of nEGFR in oral carcinogenesis. Our results point to the importance of identifying molecular markers that help us to identify the size of genetically altered and apparently healthy oral cavity mucosa and to distinguish high-risk patients with premalignant and malignant changes, which could have implications for changing the current treatment approach for these patients.

## Figures and Tables

**Table 1:** Demographic data of subjects included in the study.

Group of Subjects	Age (Years)	Gender	Number of Subjects
Control group with healthy oral mucosa	56.56 ± 11.97 54.28 ± 12.22 52.7 ± 11.84	♂ 32 ♀ 27	59
Patients with premalignant changes	64.22 ± 14.35 64.6 ± 9.92 63.9 ± 17.46	♂ 23 ♀ 27	50
Patients with Oral squamous cell carcinoma	55 ± 10.91 56 ± 10.87 54 ± 11.22	♂ 35 ♀ 17	52

**Table 2:** Associations of expression of investigated molecular biomarkers with clinical data and pathohistological features of tumors.

Molecular Biomarker	Ki-67			p53			Cyclin D1			ABCG2			nEGFR			mEGFR		
IHC scoring	≤30%	> 30%		0/+	++		0/+	++		0/+	++/+++		0/+	++/+++		0/+	++/+++	
Subject number	n = 30	n = 22		n = 9	n = 43		n = 9	n = 43		n = 9	n = 43		n = 22	n = 30		n = 19	n = 33	
harmful habits																		
none	0	8	χ^2^ = 0.359, *p* = 0.55	3	9	χ^2^ = 1.46, *p* = 0.48	3	10	χ^2^ = 2.20, *p* = 0.33	4	9	χ^2^ = 0.28, *p* = 0.59	7	8	χ^2^ = 9.71, *p* = 0.002	6	7	χ^2^ = 1.85, *p* = 0.60
alcohol abuse	18	15		9	25		3	16		4	15		8	11		6	13	
smoking	8	17		8	22		6	33		5	34		17	22		13	26	
both factors	8	15		8	22		2	15		3	14		8	11		6	13	
TNM disease stage																		
I	9	5	χ^2^ = 2.02, *p* = 0.73	4	10	χ^2^ = 9.76, *p* = 0.28	2	12	χ^2^ = 5.48, *p* = 0.70	4	10	χ^2^ = 4.67, *p* = 0.32	2	12	χ^2^ = 14.58, *p* = 0.26	4	10	χ^2^ = 6.66, *p* = 0.88
II	7	5		4	8		2	10		3	9		8	4		5	7	
III	6	4		1	9		1	9		0	10		4	6		3	7	
IV A	5	7		7	5		4	8		2	10		5	7		5	7	
IV B	3	1		2	2		0	4		0	4		3	1		2	2	
IV C	0	0		0	0		0	0		0	0		0	0		0	0	
histological tumor grade																		
1	18	10	χ^2^ = 1.08, *p* = 0.58	9	19	χ^2^ = 3.16, *p* = 0.53	6	22	χ^2^ = 3.47, *p* = 0.48	6	22	χ^2^ = 1.24, *p* = 0.53	14	14	χ^2^ = 6.2, *p* = 0.40	10	16	χ^2^ = 5.93, *p* = 0.43
2	10	10		7	13		3	17		3	17		6	14		6	14	
3	2	2		2	2		0	4		0	4		2	2		3	1	
lymphovascular invasion																		
absent	24	16	*p* = 0.55 *	14	26	*p* = 0.53 *	7	33	*p* = 0.85 *	9	31	*p* = 0.07 *	18	22	*p* = 0.88 *	15	25	*p* = 0.78 *
present	6	6		4	8		2	10		0	12		4	8		4	8	
perineural invasion																		
absent	16	12	*p* = 0.93 *	9	19	*p* = 0.02 *	6	22	*p* = 0.54 *	8	20	*p* = 0.02 *	12	16	*p* = 0.25 *	10	18	*p* = 0.80 *
present	14	10		9	15		3	21		1	26		10	14		9	15	
extranodal extension																		
absent	26	20	*p* = 0.64 *	15	31	*p* = 0.67	9	37	*p* = 0.49 *	9	37	*p* = 0.23	18	28	*p* = 0.5 *	18	19	*p* = 0.25 *
present	4	2		3	3		0	6		0	6		4	2		2	4	
disease progression																		
no	24	16	*p* = 0.54 *	12	28	χ^2^ = 1.59, *p* = 0.66	8	32	*p* = 0.62 *	9	31	χ^2^ = 1.59, *p* = 0.07	16	21	*p* = 0.51 *	14	26	*p* = 0.66 *
yes	6	6		6	6		1	11		0	12		6	6		5	7	
HPV status																		
negative	28	22	*p* = 0.22 *	16	34	*p* = 0.11 *	8	42	*p* = 0.38 *	9	41	*p* = 0.51 *	21	26	*p* = 0.88 *	17	33	*p* = 0.19 *
positive	2	0		2	0		1	1		0	2		1	1		2	0	
second primary tumor																		
no	27	16	*p* = 0.11 *	17	26	*p* = 0.24 *	7	36	*p* = 0.75 *	8	35	*p* = 0.58 *	18	23	*p* = 0.35 *	16	27	*p* = 0.87 *
yes	3	6		1	8		2	7		9	0		4	3		3	6	

* Fisher’s exact test.

## Data Availability

All data generated or analyzed during this study are included in this published article.
